# Systemic Air Embolism Following CT-Guided Percutaneous Lung Procedures: An Imaging Analysis of Divergent Neurological Outcomes

**DOI:** 10.3390/diagnostics16132037

**Published:** 2026-06-30

**Authors:** Shuo Liang, Dan Li, Zhongyu Liu, Hong Zhang

**Affiliations:** 1 Department of Radiology, Chest Hospital, Tianjin University, Tianjin 300222, China; movingspirit@163.com; 2 Clinical School of Thoracic, Tianjin Medical University, Tianjin 300070, China; 18348908211@163.com (D.L.); 13697893993@163.com (Z.L.)

**Keywords:** systemic air embolism, cerebral air embolism, computed tomography, lung biopsy, cortical laminar necrosis

## Abstract

Systemic air embolism (SAE) is a rare but potentially catastrophic complication of computed tomography (CT)-guided percutaneous lung procedures, with reported incidence ranging from 0.02% to 0.4%. Despite its low frequency, SAE can result in severe neurological impairment or death, yet the factors that determine divergent clinical outcomes remain poorly characterized. We present two cases with contrasting neurological sequelae to elucidate the imaging spectrum and potential prognostic determinants of SAE. In Case 1, a 60-year-old man developed SAE after CT-guided fiducial marker placement for a 7-mm pure ground-glass nodule and achieved full clinical recovery. In Case 2, a 68-year-old man developed SAE following CT-guided percutaneous transthoracic needle biopsy of a 16-mm solid nodule and progressed to persistent semi-comatose status with cortical laminar necrosis. These cases illustrate the heterogeneous neurological outcomes of SAE and underscore the critical role of early post-procedure CT in detecting intravascular gas. Several factors may contribute to divergent outcomes, including procedural technique, nodule characteristics, and the extent and distribution of intracardiac and intracranial air. The absence of hyperbaric oxygen therapy in both patients further highlights the importance of prompt recognition and supportive management. Radiologists and interventionalists must maintain vigilant post-procedural surveillance to facilitate timely diagnosis and optimize patient outcomes.

**Figure 1:**
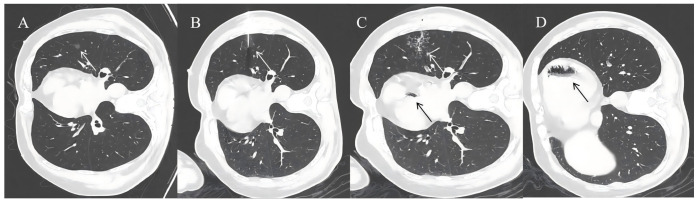
A 60-year-old male patient with no prior medical history. (**A**) Unenhanced chest CT revealed a pure ground-glass nodule (pGGN) in the anterior segment of the left upper lobe, measuring approximately 7.0 × 6.0 mm (white arrow). (**B**) Given that pGGNs are notoriously difficult to localize precisely under video-assisted thoracoscopic surgery (VATS), preoperative CT-guided placement of a metallic fiducial marker was indicated (white arrow). (**C**) Following marker deployment, ill-defined patchy opacification was observed surrounding the target lesion, consistent with procedural hemorrhage (white arrow), while intravascular gas was identified at the aortic root (black arrow). (**D**) CT image demonstrated the intravascular gas extending into the left pulmonary vein, the apex of the left ventricle, and its lateral wall (black arrow).

**Figure 2:**
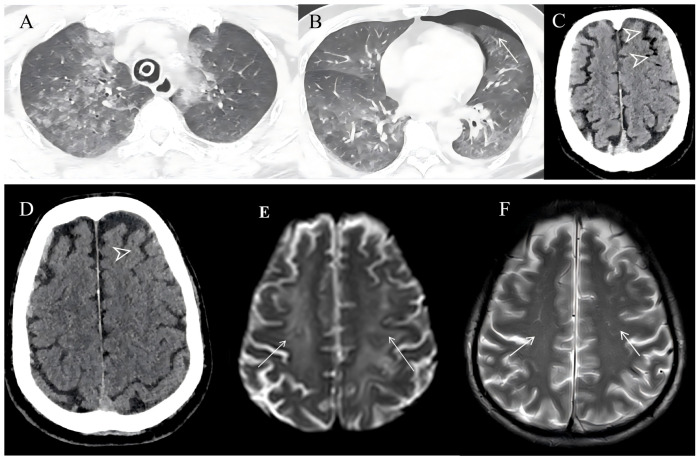
Immediately after the procedure, upon assuming an upright position, the patient suddenly lost consciousness and began expectorating fresh blood. Emergency endotracheal intubation was performed in the CT suite, and cranial and chest CT scanning was undertaken without delay. (**A**) Chest CT demonstrated bilateral, diffuse patchy ground-glass opacities consistent with multifocal pulmonary hemorrhage. (**B**) A left-sided pneumothorax was evident (white arrow). (**C**) Unenhanced head CT showed gas distributed along the cerebral sulci of the left frontal lobe (white arrowhead). (**D**) After transfer to the intensive care unit and initiation of mechanical ventilatory support, follow-up head CT performed one day later revealed partial resolution of the intracranial gas in the left frontal lobe (white arrowhead). (**E**) Brain MRI diffusion-weighted imaging obtained on day 8 showed multiple acute ischemic foci scattered throughout both cerebral hemispheres (white arrow). (**F**) Repeat brain MRI on day 42 demonstrated partial regression of the bilateral hemispheric ischemic lesions (white arrow). The patient subsequently underwent wedge resection of the lingular segment of the left upper lobe; histopathologic examination confirmed a diagnosis of minimally invasive adenocarcinoma.

**Figure 3:**
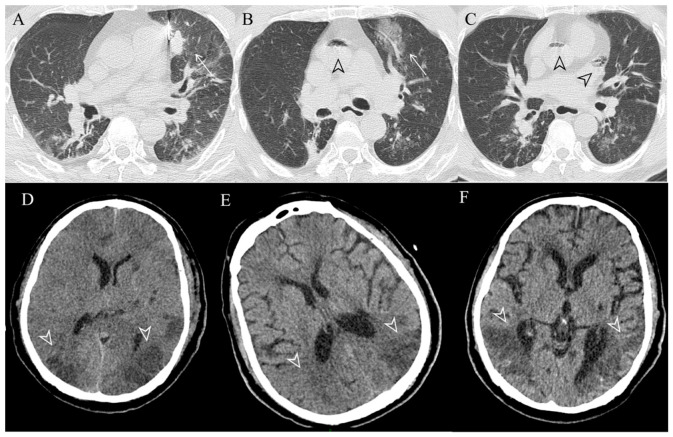
A 68-year-old male with a soft-tissue density nodule measuring approximately 16 × 10 mm in the lingular segment of the left upper lobe, identified incidentally on low-dose chest CT screening. (**A**) To establish a tissue diagnosis, CT-guided percutaneous transthoracic needle biopsy (PTNB) was performed. The biopsy needle was positioned at the medial margin of the nodule, in close proximity to the main pulmonary artery (white arrow). (**B**) Immediate post-procedure CT following needle withdrawal revealed patchy ground-glass opacification at the biopsy site in the left upper lobe lingular segment, consistent with focal hemorrhage (white arrow), and intravascular gas within the ascending aorta (black arrowhead). (**C**) Intravascular gas was further demonstrated in the ascending aorta and the left atrial appendage (black arrowhead). At this juncture, the patient abruptly lost consciousness, became unresponsive to verbal stimuli, and developed drooling from the oral commissure. Emergency resuscitation was initiated immediately. Arterial blood gas analysis revealed: pH 7.27, pCO_2_ 55.6 mmHg, pO_2_ 68.1 mmHg, SaO_2_ 88.3%, and lactate 5.6 mmol/L. Resuscitative measures included endotracheal intubation with mechanical ventilation, oropharyngeal suctioning, bedside bronchoscopy with suctioning, ice-cap application for cerebral protection, and intravenous sodium bicarbonate (5%, 50 mL) for the correction of metabolic acidosis. (**D**) Head CT performed two days later revealed multiple hypodense areas (white arrowheads) in the peritrigonal white matter of both lateral ventricles, consistent with acute cerebral infarcts. (**E**) After nine days of continued antimicrobial therapy, temperature management, mechanical ventilation via endotracheal tube, and supplemental oxygen, repeat head CT showed partial resolution of the bilateral peritrigonal hypodensities, suggesting a decrease in the extent of cerebral infarction (white arrowheads). (**F**) Head CT on day 21 post-biopsy, however, revealed interval progression of the bilateral peritrigonal hypodense lesions, with the development of multiple linear hyperdensities within the affected parenchyma, indicative of cortical laminar necrosis (white arrowheads). Clinically, the patient remained in a state of unresponsive wakefulness, exhibiting only minimal, occasional eyelid movement upon auditory stimulation. Physical examination showed a supple neck, with symmetric pupils equally reactive to light. With a heat-and-moisture exchanger in place, percutaneous oxygen saturation remained at a nadir of 90%, with documented improvement following endotracheal suctioning, indicating preserved spontaneous respiratory effort with continued dependence on the artificial airway for pulmonary toilet. Systemic air embolism (SAE) remains one of the most feared complications of CT-guided percutaneous lung procedures, with reported incidence ranging from 0.02% to 0.4% for transthoracic needle biopsy and up to 0.5% for hook-wire localization [1]. Its pathophysiology involves three main mechanisms: direct introduction of air into a pulmonary vein, creation of a bronchovenous fistula, and transpulmonary passage through disrupted alveoli [2]. Our two cases illustrate distinct pathways: in Case 1, hook-wire placement likely created a bronchovenous fistula with air entry exacerbated by positional change; in Case 2, respiratory acidosis and forceful ventilatory efforts promoted air entry [3,4]. Coughing and Valsalva maneuvers can transiently reverse bronchovenous gradients, allowing air entry [5]. The imaging evolution traces contrasting trajectories. In Case 1, scattered ischemic foci on day 8 MRI reflected microembolic occlusion with subsequent reperfusion, and near-complete resolution by day 42 correlated with full clinical recovery [6]. Case 2 followed a different course: bilateral peritrigonal infarcts progressed to cortical laminar necrosis (CLN) by day 21, signifying irreversible injury [7]. CLN represents selective necrosis of cortical layers III and V, typically appearing approximately two weeks after infarction; its early emergence in Case 2 reflects the unique pathophysiology of gas embolism, in which direct endothelial injury accelerates parenchymal damage. The persistence of semi-comatose status in Case 2, contrasted with complete recovery in Case 1, suggests that extensive cerebral infarction, early CLN, and persistent coma may be associated with poor neurological outcome. These cases carry important clinical implications. First, immediate post-procedure CT surveillance enables prompt SAE recognition [5]. Second, SAE prognosis is modulated by embolic volume, distribution, hemodynamic compromise, and cerebrovascular reserve [8]. Third, institutional protocols for SAE management—including immediate recognition, hemodynamic resuscitation, 100% oxygen, Durant maneuver, and HBOT pathways—should be standardized [9]. Prevention strategies remain paramount: coaxial techniques with continuous needle tip visualization, avoidance of biopsy through aerated lung when feasible, and strict patient instruction to avoid coughing or Valsalva maneuvers during needle deployment represent the most effective risk-reduction measures. The divergent outcomes presented here serve as a compelling reminder that even within the same institution and without differential access to advanced therapies, the spectrum of SAE extends from fully reversible neurological insult to devastating permanent injury—distinctions governed by the interplay of procedural, anatomical, and physiological variables.

## Data Availability

No new data were created or analyzed in this study.

## Abbreviations

The following abbreviations are used in this manuscript:

SAE	Systemic air embolism
CLN	Cortical laminar necrosis
CT	Computed tomography
GGN	Ground-glass nodule
HU	Hounsfield units
MRI	Magnetic resonance imaging
pCO_2_	Partial pressure of carbon dioxide
pGGN	Pure ground-glass nodule
pO_2_	Partial pressure of oxygen
PTNB	Percutaneous transthoracic needle biopsy
SaO_2_	Oxygen saturation
VATS	Video-assisted thoracoscopic surgery
